# Deterioration Effects and Microscopic Mechanisms of Solidified/Stabilized Red Mud by CGFPA Binders Under Freeze–Thaw Cycles

**DOI:** 10.3390/ma18030592

**Published:** 2025-01-28

**Authors:** Lijun Yan, Junjie Yang, Yalei Wu, Fengmin Li

**Affiliations:** 1 Key Laboratory of Marine Environment and Ecology, Ministry of Education, Ocean University of China, Qingdao 266100, China; yanlijun313@126.com (L.Y.); jjyang@ouc.edu.cn (J.Y.); lifengmin@ouc.edu.cn (F.L.); 2 College of Environmental Science and Engineering, Ocean University of China, Qingdao 266100, China

**Keywords:** red mud, solid waste, solidification/stabilization, freeze–thaw cycles, deterioration, microscopic mechanisms

## Abstract

Red mud is a kind of solid waste in the production process of the aluminum industry. The long-term stockpiling of red mud not only occupies a large amount of land but also causes environmental pollution. In order to improve the strength, reduce the alkalinity and toxicity of red mud, and study its durability under freeze–thaw cycles, CGFPA binders, whose components were calcium carbide residue, ground granulated blast furnace slag, fly ash, phosphogypsum, and graphene, were adopted to solidify/stabilize red mud in this paper. The effects and the mechanism of freeze–thaw cycling on the unconfined compressive strength, pH value, and toxic leaching of the solidified/stabilized red mud was investigated. The micro-mechanism was analyzed by XRD, SEM-EDS, and FT-IR. The results of the study showed that the mass, unconfined compressive strength, and pH of the solidified/stabilized red mud decreased gradually with an increase in the number of freeze–thaw cycles, while the leaching concentration of pollutants increased gradually. The rate of loss of unconfined compressive strength satisfies an exponential function with the number of cycles, and the logarithm of pollutant concentration satisfies a linear relationship with the number of cycles. The cumulative loss of mass was 6.7%, 5.4%, 3.6%, and 3.3%, and the cumulative loss of unconfined compressive strength was 50.6%, 47.5%, 32.2%, and 25.3%, and the pH value was reduced to 9.42, 9.54, 9.80, and 9.92, respectively, after 10 freeze–thaw cycles at binder mixing ratios of 15%, 20%, 25%, and 30%, while the leaching concentrations of Cu, Zn, Cr, Ni, As, Pb, and Cd increased from 7.4 μg/L, 87.2 μg/L, 5.2 μg/L, 7.0 μg/L, 6.9 μg/L, 3.7 μg/L, and 0.7 μg/L to 17.5 μg/L, 123.5 μg/L, 10.2 μg/L, 15.7 μg/L, 11.4 μg/L, 5.6 μg/L, and 4.9 μg/L, respectively, under the condition of a 30% incorporation ratio. The gelling products generated by the hydration reaction of the binders were mainly C-S-H, C-A-S-H, C-A-H, AFm, etc. Under the action of freeze–thaw cycles, the lattice-like structure of the solidified/stabilized red mud was damaged, resulting in a decrease in its unconfined compressive strength and an increase in pollutant leaching concentration. The research results can provide a theoretical basis for the use of red mud in permafrost regions.

## 1. Introduction

Red mud is an industrial solid waste produced after the production of alumina or aluminum hydroxide from bauxite ores [1], which is so-named because of its high iron oxide content and its similarity in appearance to red clay. Red mud is divided into Bayer red mud (hereinafter referred to as red mud), sintered red mud, and combined red mud according to the alumina production process. The Bayer method production process is that since bauxite contains impurities, it needs to be treated to produce a purer form of alumina, Al_2_O_3_. This is achieved by adding blended and milled bauxite (for a stabilized feed) along with sodium hydroxide (NaOH) to a pressure vessel at 145–265 °C, c. 3.5 megapascals (MPa) to form a hydrated NaAl(OH)_4_ slurry, followed by filtration to remove impurities and insoluble compounds from the reaction (e.g., coarse particles and sodium-aluminosilicate precipitates) and chemically treated to remove other soluble compounds to form a supersaturated sodium alumina solution. Water is then removed from the Al(OH)_3_ crystals to give anhydrous alumina [2]. The waste sludge produced in this process is the Bayer red mud. Because it is relatively simple, does not require a high-temperature calcination process, and is suitable for processing higher-grade bauxite ores, more than 95% of the global aluminum industry uses the Bayer method [3]. However, the preparation of alumina by the Bayer method requires the use of sodium hydroxide to treat the bauxite ore during the production process, resulting in an alkaline red mud with a pH value of typically 10–14 [3,4,5,6,7,8]. In addition, red mud contains a wide range of contaminants. In addition, radioactive elements ^232^Th, ^226^Ra, and ^40^K are present in concentrations [9] that are much higher than the world average of radionuclide concentrations in construction materials (^232^Th = 50 Bq/kg, ^226^Ra = 50 Bq/kg, ^40^K = 500 Bq/kg), with concentrations of ^232^Th and ^226^Ra up to 5–7 times the average [10,11]. Red mud is recognized as hazardous waste in countries such as Portugal and India [12,13]. The leaching concentrations of 16 hazardous components of red mud from eight aluminum companies in China are lower than the identification standard of “Hazardous Waste Identification Standard Leaching Toxicity Identification” [14]. According to the Catalog of Solid Waste Classification and Codes issued by the Ministry of Ecology and Environment in 2024, red mud is classified as general industrial solid waste, and it is mainly stockpiled currently [15]. By the end of 2022, the accumulated stockpile of red mud in China exceeded 1.70 billion tons [16]. Although red mud is not a hazardous waste, high alkalinity and a variety of pollutants are the main problems that limit its utilization. Long-term stockpiling makes the pollutants in red mud migrate through precipitation, irrigation, and groundwater runoff, leading to soil and groundwater contamination [17], and there are safety hazards such as landslides in landfills [18,19,20].

The main methods for the harmless treatment of red mud are physical wrapping [21], chemical reagent improvement [13], and solidification/stabilization [22,23,24,25,26,27]. Among them, solidification/stabilization is widely used due to the advantages of lower cost, convenient construction, and good engineering performance after treatment [28,29]. Cement is the most commonly used binder for solidification/stabilization [30,31]. Studies have shown that cement has an obvious inhibitory effect on the leaching of hazardous substances in red mud [22], and after solidified/stabilized by cement, the leaching concentrations of As, Cd, Cr, Pb, Cu, and Ni are lower than 0.01 mg/L, and there is no risk of environmental safety [23]. The Cr removal rate reaches 95.6–99.3%, the As removal rate reaches 99.9%, and the fluoride removal rate reaches 84.9% [22,24]. If red mud is solidified/stabilized by cement and fly ash, the leaching concentrations of heavy metals such as As, Cd, Cr, and Pb in the leach solution can be lower than the standard requirements [25,26], in which the removal rates of As, Hg, Pb, Cr, and Cu reach 96.6%, 96.9%, 99.6%, 93.6%, and 82.7%, respectively [27]. However, the production of cement brings problems such as high energy consumption, high pollution, and the consumption of large amounts of non-renewable resources; therefore, it is necessary to find a low-carbon and efficient binder to replace cement.

Currently, there are all-solid-waste binders prepared using alkaline solid wastes such as calcium carbide residue and alkali residue as alkali activator, active silica–aluminum solid wastes such as ground granulated blast furnace slag, fly ash, steel slag, and rice husk ash as pozzolanic materials, and phosphogypsum as supplementary materials. These can effectively reduce heavy metals such as Ni, Zn, Cu, Cd, Pb, etc., and increase the strength of heavy-metal-polluted soil [32,33,34,35,36,37,38,39,40,41,42,43,44,45,46,47,48]. In addition, graphene has a high specific surface area and the surface is rich in active oxygen-containing functional groups, which can be physically adsorbed and covalently reacted; at the same time, it has high hydrophilicity and negative charge density and is considered to be a highly efficient adsorbent for adsorption of various heavy metal ions [49]. The addition of 0.05–0.1% graphene to cementitious materials can effectively improve their unconfined compressive strength [50]. The group’s previous research showed that CGFPA binders prepared with calcium carbide residue (C) as an alkali exciter, ground granulated blast furnace slag (G) and fly ash (F) as a volcanic ash material, phosphogypsum (P) as auxiliary material, and graphene (A) as an external admixture when used for solidifying/stabilizing red mud, could effectively increase the unconfined compressive strength of the red mud, and reduce the pH value and pollutant concentration. When the mixing ratio of the binder was 30%, the optimal total water content ratio was 1.4, the curing age was 90 d, the unconfined compressive strength reached 6.9 MPa, the pH value was reduced to 9.47, and the leaching concentrations of Cu, Zn, Cr, Ni, As, Pb, and Cd were reduced to 7.4 μg/L, 87.2 μg/L, 5.2 μg/L, 7.0 μg/L, and 6.9 μg/L, 3.7 μg/L, and 0.7 μg/L, respectively [51].

The properties of red mud vary with hydrogeological and climatic conditions [52]. It has been shown that freeze–thaw cycling increases the porosity of solidified/stabilized red mud [52] and decreases its mechanical properties [53,54]. For example, if lime and fly ash were used to solidify/stabilize red mud, the unconfined compressive strength was reduced from 3.4 MPa to 3.2 MPa after freeze–thaw cycles, with a strength loss of 5.88% [55], while the unconfined compressive strength of solidified/stabilized red mud with ground granulated blast furnace slag was reduced from 9.97 MPa to 6.89 MPa after five freeze–thaw cycles, with a strength loss of 30.89% [56]. The solidification/stabilization of red mud using cement, lime, and phosphogypsum reduced the unconfined compressive strength to 2.63–3.70 MPa after five freeze–thaw cycles, and the strength loss rate reached 54.82–79.79% [57,58]. Therefore, it is necessary to study the freeze–thaw cycle resistance of solidified/stabilized red mud to guarantee its reliability during service [59].

Since the properties of solidified/stabilized red mud are easily changed under the influence of environmental conditions, affecting its service life, this paper focuses on the change rule of the mechanics, chemistry, and leaching toxicity of solidified/stabilized red mud with the influence of the number of freeze–thaw cycles. In this paper, CGFPA was adopted to solidify/stabilize red mud that could be used in permafrost regions through the freeze–thaw cycle. The unconfined compressive strength, acidity, and alkalinity were studied, alongside toxicity leaching, and the freeze–thaw cycle effect on the strength of solidified/stabilized red mud, pH value, pollutant leaching concentration influence law. At the same time, the micro-mechanism was studied using XRD, SEM-EDS, and FT-IR analysis of the micro-mechanism. The research results can provide a theoretical basis for the use of red mud in permafrost region.

## 2. Materials and Methods

### 2.1. Test Materials

**Figure 1:**
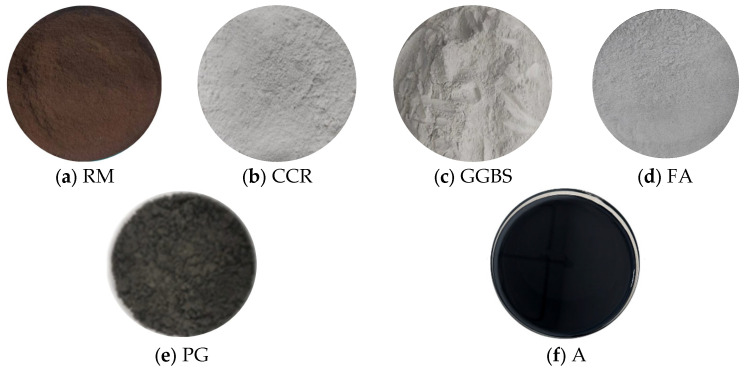
Red mud and binder components of CGFPA.

**Figure 2:**
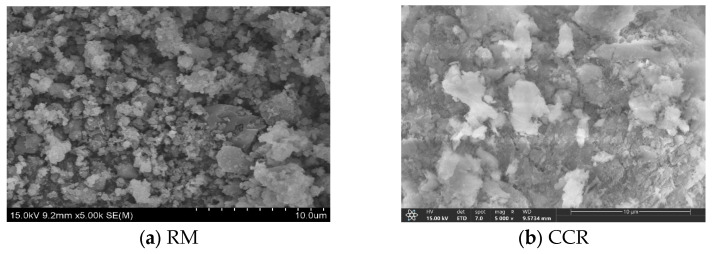

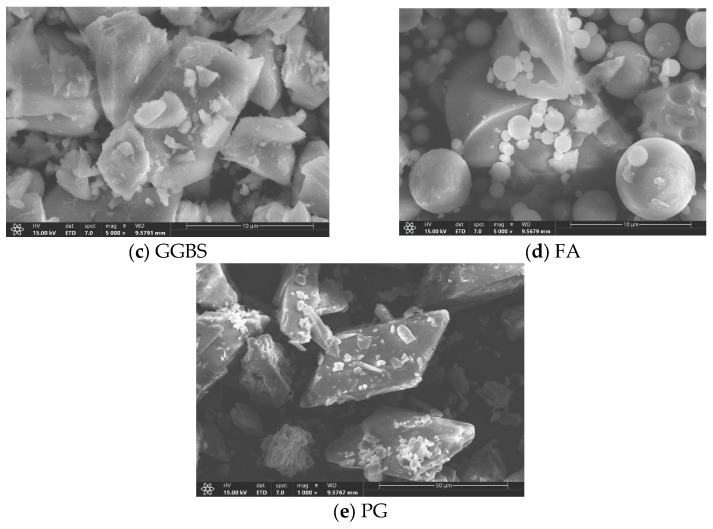
SEM of red mud and CGFPA.

The red mud used in the test was the solid waste generated by the Aluminum Corporation of China Guangxi Branch-Bayer method red mud (RM). A photograph of the red mud is shown in Figure 1a and the SEM pattern is shown in Figure 2a. The gradation curve is shown in Figure 1, the basic physical properties are shown in Table 1, and the chemical composition is shown in Table 2. The pH value of the red mud is 10.01. The leaching concentrations of common pollutants in red mud measured by ICP-MS are shown in Table 3, in which the leaching concentrations of four pollutants, Ni, As, Pb, and Cd, were higher than the Class III water quality standard in the Groundwater Quality Standard [60].

**Figure 3:**
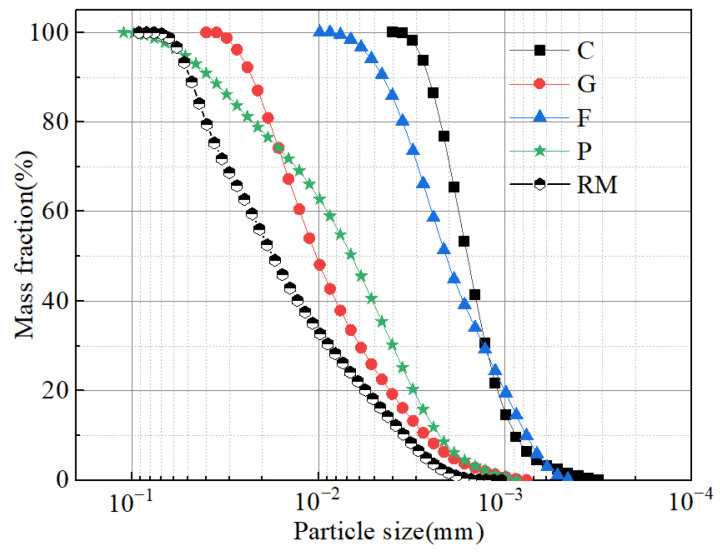
Grading diagram of the materials.

The binder used in the test is CGFPA binder. A photograph of the binder is shown in Figure 1b–f and the SEM pattern is shown in Figure 2b–e. The particle size grading curve of the components is shown in Figure 3. In the binder, the calcium carbide residue is taken from the waste residue after the production of acetylene by Qingdao Industrial Gases Company Limited in Shandong Province; the ground granulated blast furnace slag is taken from the water quenching of S 105 granulated blast furnace slag by an iron and steel plant in Jinan, Shandong Province; the fly ash is taken from the I level fly ash of an iron and steel plant in Qingdao, Shandong Province; the phosphogypsum is taken from the industrial by-products of a chemical plant in Linyi, Shandong Province. The pH of the binder is 4.1. As an auxiliary material to reduce the pH value of red mud, the external dopant graphene supplied by a company in Xiamen is used. Since graphene is a single atomic plane of graphite, it is usually considered to be a monolayer hexagonal honeycomb two-dimensional planar structure with a thickness of only one carbon atom, i.e., 0.335 nm. It was therefore not included in the particle grading curve.

### 2.2. Test Formulas

In this paper, the total water content ratio (Equation (1)) is used to characterize the total degree of water content of the solidified/stabilized specimens before mixing.

(1) $$\alpha_{wt}=\frac{w_{nt}}{w_{L}}$$

where α*_wt_* is the total water content ratio, dimensionless; *w_nt_* is the total initial water content, which is the ratio of all water in the solidified/stabilized specimen before mixing (including water in the soil, water in the admixture, and added water) to the mass of the dry soil, %; and *w_L_* is the liquid limit of the soil, %.

The cumulative rate of mass loss of the specimen is calculated according to Equation (2).

(2) $$K_{m}=\frac{m_{0}-m_{n}}{m_{0}}\times 100$$

where *K_m_* is the cumulative rate of mass loss, %; *m_n_* is the mass of the specimen after the nth cycle, g; and *m*_0_ is the initial mass of the specimen before the freeze–thaw cycles, g.

The cumulative rate of loss of strength of the specimen is calculated according to Equation (3).

(3) $$K_{q}=\frac{q_{u0}-q_{un}}{q_{u0}}\times 100$$

where *K_q_* is the cumulative rate of strength loss, %; *q_u_*_0_ is the strength of the specimen before cycling, kPa; and *q_un_* is the strength of the specimen after the nth cycle, kPa.

The smaller *K_q_* is, the more resistant the specimen is to freeze–thaw cycles.

Similar to the rate of loss of mass and rate of loss of strength, the cumulative rate of decrease in the pH value of the specimen was calculated according to Equation (4)

(4) $$K_{pH}=\frac{pH_{0}-pH_{n}}{pH_{0}}\times 100$$

where, *K_pH_* is the cumulative reduction rate of pH, %; *pH*_0_ is the pH value of the specimen before freeze–thaw cycling, dimensionless; *pH_n_* is the pH value of the specimen after n times freeze–thaw cycles, dimensionless.

### 2.3. Test Program

According to the results of a large number of tests, the lowest value of the strength of cured soil generally occurs at about 10 freeze–thaw cycles, after which it will be gradually stabilized [61], so the number of freeze–thaw cycles is set in this program as 2, 4, 6, 8 and 10 times. The test program is shown in Table 4.

### 2.4. Test Process

**Figure 4:**
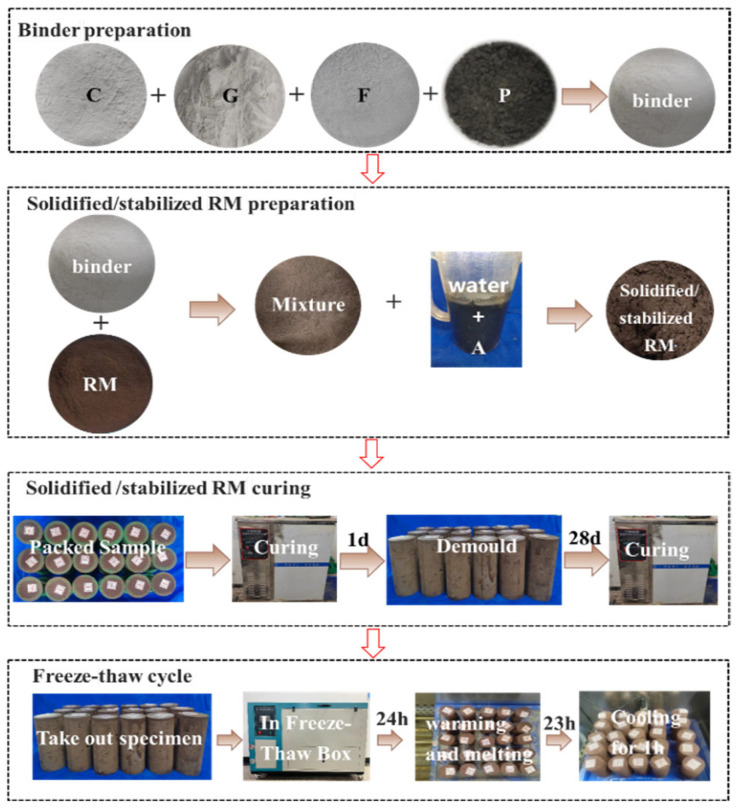
Process of test.

The test process can be summarized as follows. Firstly, according to the mixing ratio of the CGFP binder, CCR, GGBS, FA, and PG were weighed in the ratio of 4:4:2:1, which were mixed and stirred well to obtain CGFP binders. Secondly, the graphene (A) slurry was taken with the weights of 0.1% of CGFP binder and diluted with water according to the water content (water content with graphene). Thirdly, the slurry was poured into the mixture of red mud and binder and mixed well, and the mixture was filled into a standard test mold with a diameter of 50 mm and a height of 100 mm in 3 layers. Each layer was scraped and the mold was filled. There were three replicates of each sample, with a mass error that did not exceed 5 g. Fourthly, the prepared specimen was put into a standard curing box for standard curing at a temperature of 20 ± 5 °C, with a relative humidity of 95%, and demolded after 1 d. Finally, after demolding, the specimen was put into a standard curing box for up to 28 days of curing and a freeze–thaw box for the freeze–thaw cycle test. Tests of the unconfined compressive strength, acidity and alkalinity, toxicity leaching, and microscopic structure were carried out after the number of cycles was reached. The test process is shown in Figure 4.

### 2.5. Test Methods

The freeze–thaw cycle test was conducted in accordance with the ASTM-D560-03 standard of the U.S.A. [62]. The test instrument was the CLD-type automatic low-temperature freeze–thaw tester produced by Beijing Zhongke Luda Testing Instrument Co., Ltd., (Beijing, China). with external dimensions of 1900 mm × 800 mm × 1400 mm. The dimensions of the container liner were 1100 mm × 500 mm × 500 mm. The maximum thawing temperature was 30 °C and the minimum freezing temperature was −30 °C. The design temperature of the freeze–thaw cycle was from −20 °C to 20 °C. After the curing of the specimens, the specimens were put into the fully automated freeze–thaw test equipment to conduct the freeze–thaw cycle test. Firstly, the specimens were put into the freezing chamber at −20 °C for 24 h, then warmed up to 20 °C for melting for 23 h, and then cooled down to −20 °C for 1 freeze–thaw cycle, and 0, 2, 4, 6, 8, 10 freeze–thaw cycles were carried out [63]. After the designed number of cycles, the specimens were tested for unconfined compressive strength, pollutant leaching concentration, and pH value.

The unconfined compressive strength test (UCST) is of the strain-controlled type, with the vertical displacement rate set at 1% of the height of the specimen per minute, i.e., 1 mm/min.

The acidity and alkalinity test uses specimens after the unconfined compressive strength test. The test procedure is performed with reference to reference [64]. Firstly, after the unconfined compressive strength test, samples were taken from the crushed samples. Secondly, they were dried to constant weight at 50 °C, crushed, sieved (<2 mm), and about 10.00 g of the sieved sample was mixed with 50 mL of distilled water. Thirdly, after stirring vigorously for 3 min with a magnetic stirrer and left to stand for 30 min, the filtrate was tested for pH using a Shanghai Leici PHS-3E acid meter produced by Shanghai Leici Instrument Co., Ltd., (Shanghai, China).

The leaching toxicity test of pollutants was conducted using specimens at the end of the unconfined compressive strength test. The test procedure was carried out with reference to reference [65]. Firstly, a small amount of the specimen after the end of the unconfined compressive strength test was taken, dried, pulverized, and passed through a 0.5 mm sieve. Secondly, 5.7 mL of glacial acetic acid was added to deionized water, and the volume was fixed to 1.0 L to obtain an acetic acid leaching solution with pH 2.88. Next, 5.0 g of sample was accurately weighed, according to a liquid–solid ratio of 20:1 to add the required extraction solution, placed in the shaking box at 180 r/min speed, and subjected to 18 h of shaking followed by standing for 2 h to obtain the mixture. Then, a vacuum filtration pump and a filter membrane with a pore size of 0.22 μm were used to filter the above mixture, and the filtrate was obtained. Finally, a small amount of the filtrate was placed in a beaker, and then the remaining filtrate was put into a plastic bottle, and the concentration of pollutants in the leachate was analyzed by inductively coupled plasma mass spectrometry (ICP-MS).

Tests of mineral phase composition were carried out by XRD analysis. This was performed using specimens from the UCST. After the UCST, the specimen was sampled from the hand-broken sample and dried at 50  °C until constant weight; the XRD of the sample was measured using approximately 10.00  g of crushed and sieved (<75  µm) sample. XRD measurements were performed using a Rigaku Smartlab Se X-ray instrument produced by Japan Rigaku Co., Ltd., (Tōkyō, Japan). The X-ray generator was a 40 kV, 40 mA phototube; and the target was a copper rotating anode. The scanning method employed a 2θ goniometer, accuracy 0.002°, and a scanning angle of 5–75°.

A scanning electron microscope with a built-in energy spectrometer (SEM-EDS) was used to analyze the microscopic morphology and elemental composition, and the magnification was set to 1000, 5000, and 20,000. The SEM-EDS measurements were performed using a benchtop scanning electron microscope with built-in energy spectrometer (GeminiSEM 300, manufactured by Zeiss, Jena, Germany).

Fourier Transform Infrared Spectroscopy (FT-IR) was carried out using an IRAffinity-1S FT-IR spectrometer manufactured by Shimadzu Corporation of Japan (Kyoto, Japan). Samples were first baked in an oven at 50 °C until constant weight, and then powdered, and sieved through a 45 μm sieve. The test method was the potassium bromide (KBr) compression method, and the mass ratio of KBr to sample powder was 100:1. The test wave numbers ranged from 600 cm^−1^ to 400 cm^−1^, and the respective rates were 4 cm^−1^.

## 3. Results and Discussion

### 3.1. Changes in Physical Indicators

**Figure 5:**
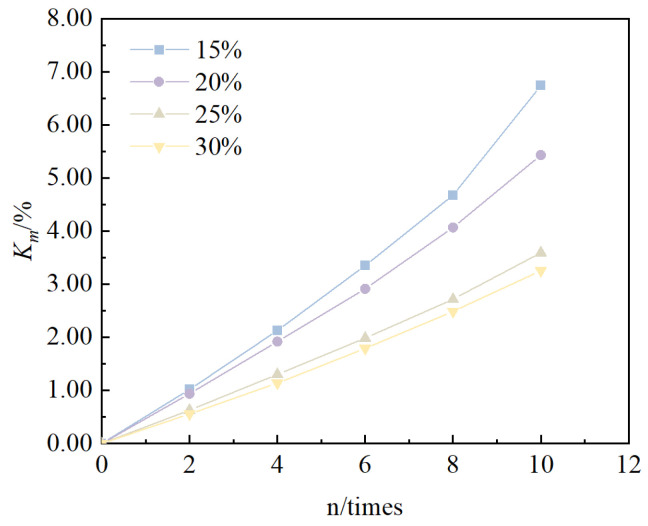
Relationship between *K_m_* and n.

The relationship between the cumulative mass loss rate of solidified/stabilized red mud and the number of cycles is shown in Figure 5. It can be seen that, under the condition of the same binder mixing ratio, the cumulative mass loss rate of solidified/stabilized red mud increases with an increase in the number of freeze–thaw cycles, which indicates that the larger the number of freeze–thaw cycles, the larger the loss in mass. However, under the condition of the same number of cycles, the cumulative mass loss rate of solidified/stabilized red mud decreases with an increase in the dosing ratio, i.e., the curve is shifted to the lower right, which indicates that the larger the mixing ratio, the lower the mass loss.

### 3.2. Changes in Mechanical Indicators

#### 3.2.1. Stress–Strain Curve

**Figure 6:**
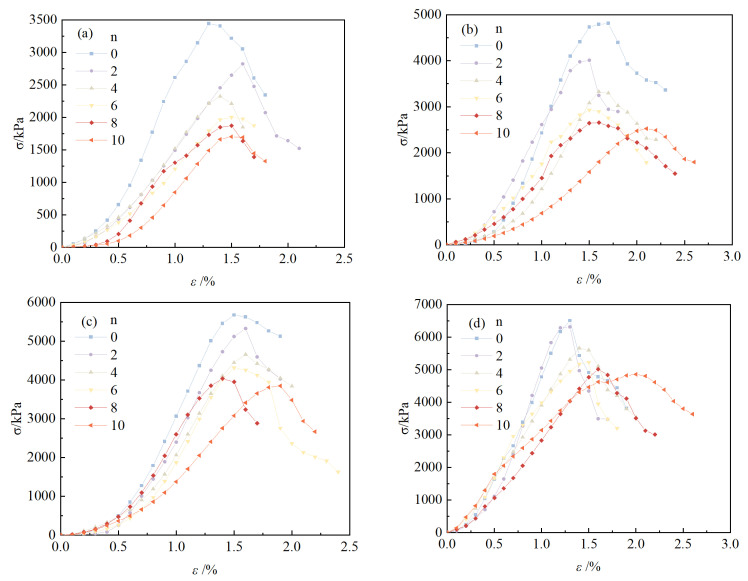
Stress–strain curves of solidified red mud with different mixture ratios. (**a**) *a_s_* = 15%; (**b**) *a_s_* = 20%; (**c**) *a_s_* = 25%; (**d**) *a_s_* = 30%.

The stress–strain curves of solidified/stabilized red mud with different mixing ratios are shown in Figure 6. As can be seen from the figure, the stress–strain curves of solidified/stabilized red mud show a more pronounced peak stress, which presents the typical brittle deformation characteristics of the strain-softening type, and its destructive strain is between 1.3% and 2.2%.

#### 3.2.2. Unconfined Compressive Strength Test

**Figure 7:**
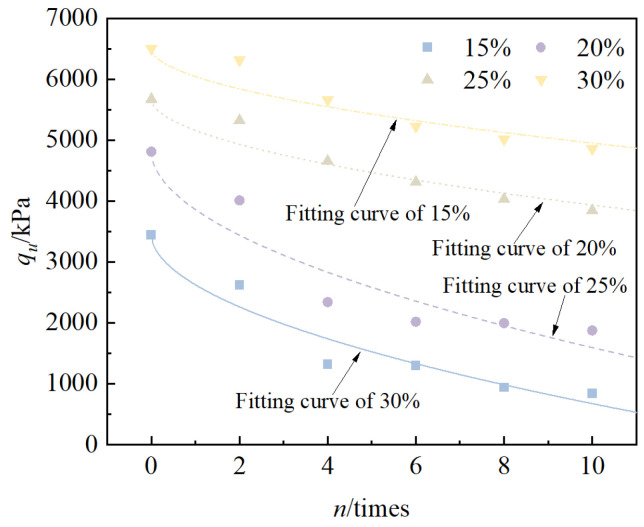
Relationship between *q_u_* and *n*.

Taking the peak stress as its unconfined compressive strength value, the relationship between the unconfined compressive strength of solidified/stabilized red mud and the number of cycles is shown in Figure 7. It can be seen from the figure that, under the condition of the same binder mixing ratio, with an increase in the number of cycles, the unconfined compressive strength of the solidified/stabilized red mud decreases gradually, and this decreasing trend decreases with an increase in the mixing ratio, i.e., the curve is more gentle. The unconfined compressive strength of the specimens decreased from 3443.2 kPa to 1843.44 kPa, 4810.3 kPa to 2525.1 kPa, 5673.0 kPa to 3847.2 kPa, and 6503.0 kPa to 4856.3 kPa under the conditions of the four binder mixing ratios, respectively, and the pattern is consistent with the model of the exponential function and can be fitted into Equation (5).

(5) $$q_{un}=q_{u0}-a\times n^{0.53}$$

where, *q_un_* is the unconfined compressive strength of the specimen after *n* times of freeze–thaw cycles, kPa; *q_u_*_0_ is the unconfined compressive strength of the specimen before freeze–thaw cycles, kPa; and *a* is the fitting parameter.

The fitting parameters are shown in Table 5, from which it can be seen that the fitting parameter *a* is positive, its range is between 457.6–948.6, and the coefficient of determination R^2^ for all mixing ratios is greater than 0.9.

**Figure 8:**
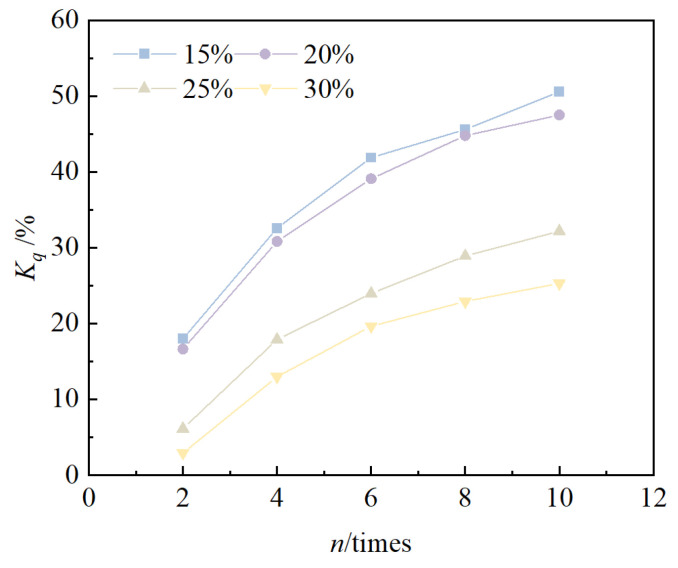
Relationship between *K_q_* and *n*.

The relationship between the cumulative loss of strength rate and the number of cycles is shown in Figure 8. From the figure, it can be seen that the cumulative loss of strength rate of solidified/stabilized red mud increases gradually with an increase in the number of freeze–thaw cycles. Under the condition of the same number of cycles, the larger the blending ratio, the lower the cumulative loss rate of strength of solidified/stabilized red mud, i.e., the curve shifts downward. After 10 freeze–thaw cycles, the cumulative loss rate of unconfined compressive strength of the specimens at 15%, 20%, 25%, and 30% mixing ratios were 50.6%, 47.5%, 32.2%, and 25.3%, respectively. This shows that the larger the mixing ratio, the smaller the cumulative loss of strength, and the stronger the freeze–thaw cycles of solidified/stabilized red mud.

According to the functional failure evaluation criteria, the solidified/stabilized red mud can be considered to have failed when the strength of the solidified/stabilized red mud is lower than 60% of the strength before the freeze–thaw cycles [52], i.e., the solidified/stabilized red mud can be considered to have failed when *q_un_* is lower than 60% of *q_u_*_0_. According to Equation (3), the number of freeze–thaw cycles at the failure of solidified/stabilized red mud is 2, 3, 16, and 26 times when the blending ratio is 15%, 20%, 25%, and 30%, respectively.

### 3.3. Changes in Chemical Indicators

**Figure 9:**
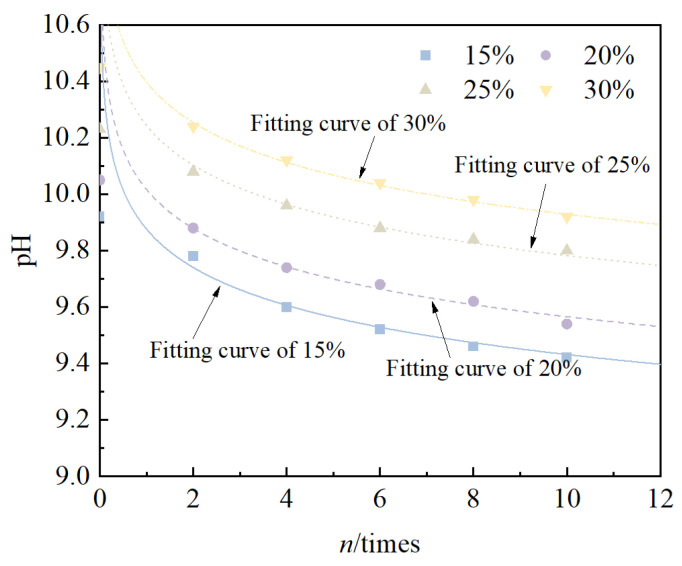
Relationship between *pH* and *n*.

The relationship between the pH value of solidified/stabilized red mud and the number of freeze–thaw cycles is shown in Figure 9. It can be seen from the figure that, under the same conditions, the pH value of the solidified/stabilized red mud gradually increased with an increase in the mixing ratio, i.e., the curve shifted upward. This is due to the fact that the content of calcium carbide residue as an alkali exciter in the binder component increases with an increase in the mixing ratio, thus leading to an increase in its pH value. Whereas, with the increase in the number of cycles, the pH value of solidified/stabilized red mud for all four mixing ratios gradually decreases, and the decreasing trend gradually becomes slower. This is consistent with the results of Wang et al. [58]. The pH value of solidified/stabilized red mud decreased from 9.92, 10.05, 10.23, 10.45 to 9.42, 9.54, 9.80, and 9.92, respectively, after 10 freeze–thaw cycles when the mixing ratios were 15%, 20%, 25%, and 30%. The pattern can be fitted as an exponential function, which can be expressed by Equation (6)

(6) $$pH_{n}=b\times n^{-0.02}$$

where *pH_n_* is the pH value of the specimen after n times freeze–thaw cycles, dimensionless, and *b* is a fitting parameter related to the type of binder and the number of cycles.

The fitting parameters are detailed in Table 6. From the table, it can be seen that the coefficients of determination are all greater than 0.95, which indicates that the formulae have a good correlation. The fitting parameter *b* increases with an increase in the mixing ratio and its value ranges from 9.93 to 10.39.

**Figure 10:**
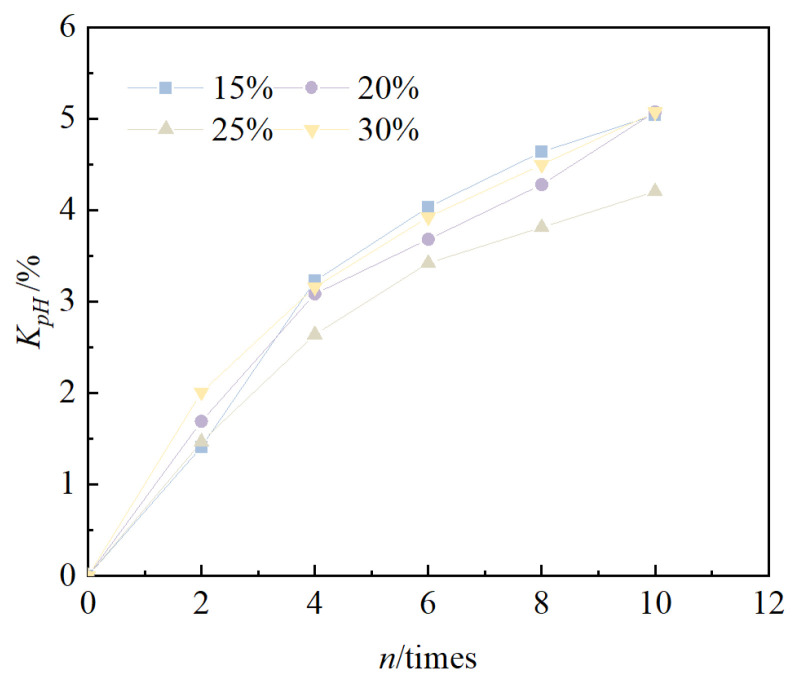
Relationship between *K_pH_* and *n*.

The relationship between the cumulative reduction rate in pH of the specimen and the number of cycles is shown in Figure 10. It can be seen that, with an increase in the number of freeze–thaw cycles, the cumulative reduction rate in pH of the solidified/stabilized red mud increases with an increase in the number of cycles, but the trend gradually becomes slower. This is due to the fact that with the freeze–thaw cycle, the pore water inside the specimen is formed into ice by the temperature, and the volume expansion damages the structure of the specimen; internal fissures continue to develop with an increase in the number of cycles, and when the sample enters into the thawing cycle, the internal ice is melted into water, and the OH^−^ flows to the outside of the specimen along with the pore water, which leads to a decrease in the concentration of OH^−^ in the specimen. However, with an increase in the number of cycles, the structure of the specimen is no longer destroyed, so the decreasing trend becomes slower.

### 3.4. Changes in Leaching Toxicity

**Figure 11:**
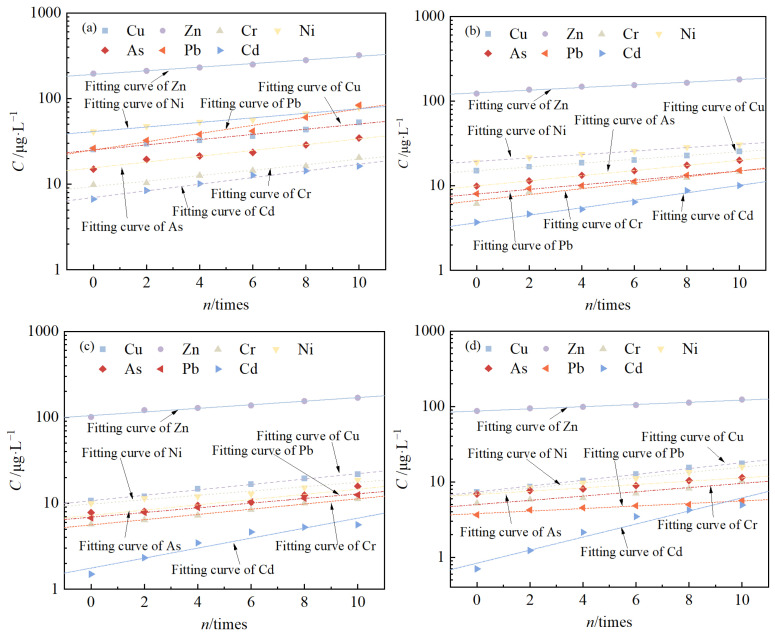
Relationship between pollutant concentration and number of cycles. (**a**) *a_s_* = 15%; (**b**) *a_s_* = 20%; (**c**) *a_s_* = 25%; (**d**) *a_s_* = 30%.

The relationship between the leaching concentration of pollutants from solidified/stabilized red mud and the number of freeze–thaw cycles is shown in Figure 11. It can be seen that the leaching concentration of Cu, Zn, Cr, Ni, As, Pb, and Cd from the solidified/stabilized red mud with four different mixing ratios increased with an increase in the number of cycles. Taking the mixing ratio of 30% as an example, the leaching concentrations of the seven pollutants increased from 7.4 μg/L, 87.2 μg/L, 5.2 μg/L, 7.0 μg/L, 6.9 μg/L, 3.7 μg/L, and 0.7 μg/L to 17.5 μg/L, 123.5 μg/L, 10.2 μg/L, 15.7 μg/L, 11.4 μg/L, 5.6 μg/L, and 4.9 μg/L respectively, after 10 freeze–thaw cycles,. The logarithm of pollutant leaching concentration and the number of cycles satisfy the linear relationship, i.e., Equation (7).

(7) lgC=k×n+c

where *C* denotes the leaching concentration of the pollutant, μg/L; *n* denotes the number of cycles, times; *k* is the slope of the straight line, and *c* is the intercept of the straight line.

After 10 freeze–thaw cycles, the pollutant As in the solidified/stabilized red mud in four binder mixing ratios were higher than the requirements of Class III water quality standards in the Groundwater Quality Standards [60], but still lower than the pollutant leaching concentrations of red mud in Table 3. This indicates that the freeze–thaw cycle effect decreases the solidifying/stabilizing effect of the binder on the red mud and increases the pollutant leaching concentration.

If the concentration limits of pollutants in the specification were adopted as the evaluation criteria for the failure of solidification/stabilization by the binder, as can be seen from Figure 9, the leaching concentration of As in the solidified/stabilized red mud had exceeded the specification limits when the mixing ratio of the binder was 15%, and the number of cycles was zero. When the mixing ratio was 20%, 25%, and 30%, after 2, 6, and 8 cycles respectively, the leaching concentration of As also exceeded the normative limit value, indicating that the solidified/stabilized red mud had failed at this time. Therefore, it can be assumed that the number of freeze–thaw cycles for solidified/stabilized red mud failing at binder mixing ratios of 15%, 20%, 25%, and 30% were 0, 2, 6, and 8, respectively, which are much lower than the above mentioned 2, 3, 16, and 26 cycles.

### 3.5. Microstructure Analysis and Degradation Mechanism

#### 3.5.1. XRD Results and Analysis

**Figure 12:**
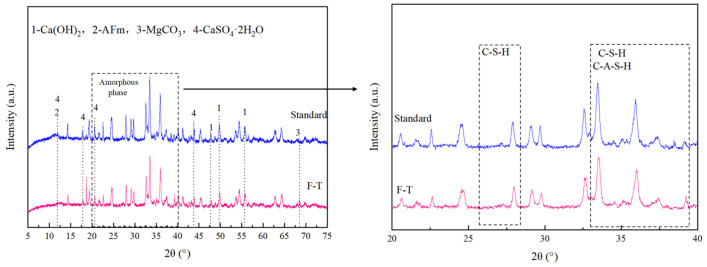
XRD patterns of solidified red mud under freeze–thaw cycle environments.

Figure 12 shows the XRD pattern of red mud solidified/stabilized by CGFPA binder after 10 freeze–thaw cycles and solidified/stabilized red mud under standard conditions. As can be seen from the figure, the products after freeze–thaw cycles are largely the same as those under standard conditions, mainly alkali-stimulated hydration products AFm, CaSO_4_·2H_2_O, MgCO_3_, C-A-H, C-S-H, C-A-S-H, Ca(OH)_2,_ and so on. Compared to the solidified/stabilized red mud under standard conditions, the diffraction peak intensity of the amorphous phase in the region of 2θ ≈ 20–40 ° was slightly reduced after 10 freeze–thaw cycles, indicating that the amorphous phase structure was partially damaged [66], which was attributed to the expansion and contraction forces generated by the freeze–thaw cycles, resulting in the formation of whiskers on the surface of the solidified/stabilized red mud and more micropores, thus destroying the gel structure of C-A-H, C-S-H, and C-A-S-H [67], leading to a decrease in their content. As these products are the main factors affecting the strength of solidified/stabilized red mud, with a decrease in their content, there is also a decrease in the strength of the solidified/stabilized red mud after freeze–thaw cycling. The increase in the MgCO_3_ peak and the decrease in the Ca(OH)_2_ peak in the figure indicate that OH^−^ reacts with CO_2_ to produce CO_3_^2−^ during the freeze–thaw cycle, which also confirms the conclusion drawn from the pH decrease in Section 2.2. The CaSO_4_·2H_2_O peak also becomes smaller after freeze–thaw cycling, which is due to the continuous loss of H_2_O in CaSO_4_·2H_2_O during freeze–thaw cycling under the influence of temperature change.

#### 3.5.2. SEM-EDS Results and Analysis

**Figure 13:**
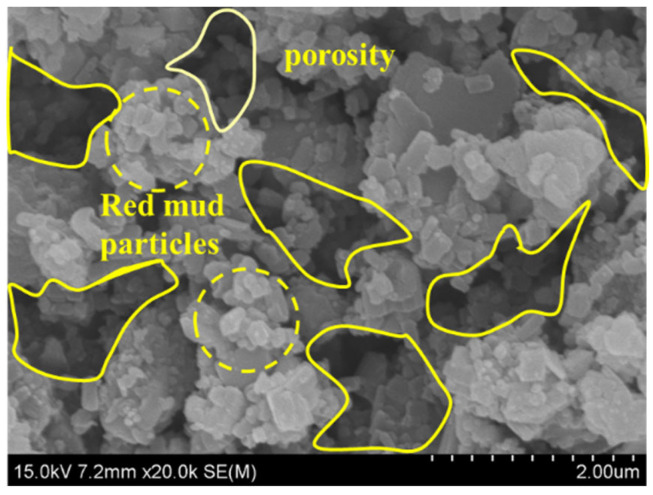
SEM of red mud.

Figure 13 shows the SEM images of red mud. As can be seen from the figure, there are a large number of fine particles and pores in the red mud, which is the main reason for the low strength of red mud.

**Figure 14:**
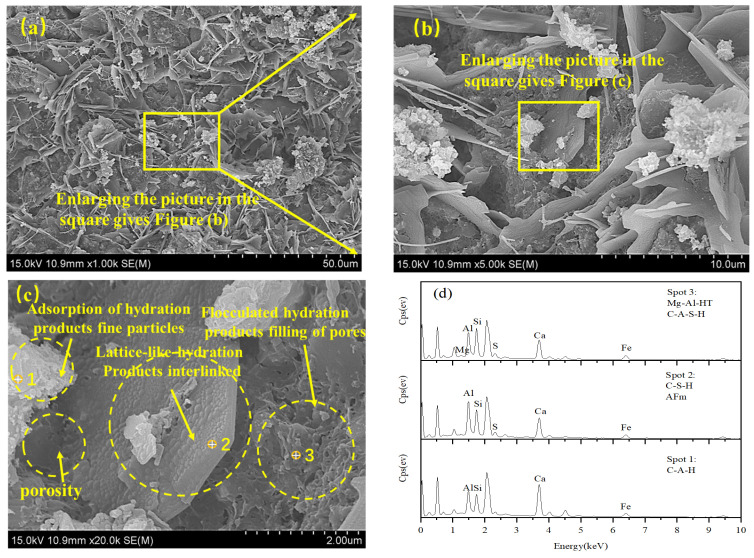
SEM-EDS of solidified/stabilized red mud. (**a**) ×1000; (**b**) ×5000; (**c**) ×20,000; (**d**) EDS.

Figure 14 is the SEM-EDS image of solidified/stabilized red mud after adding CGFPA binder and standard curing for 28 d. As can be seen from the figure, compared with red mud, a large number of grid-like hydration products were generated and connected with each other in the solidified/stabilized red mud, while there were also some hydration products gelling and adsorbing the red mud particles, which made the integrity stronger. In addition, a large number of flocculated hydration products fill the pores, making the porosity lower and the structure denser and stronger. Combined with the EDS analysis, the peaks of Al and Ca were higher at point 1, which might be C-A-H. Point 2 was a flaky and lattice-like material and contained elements such as Ca, Si, and Al, indicating that it contained AFm and C-S-H [68]. Point 3 is a flocculated material with high peaks of Mg, Al, and Si elements, indicating the presence of Mg-Al-HT and C-A-S-H. These hydration products solidified/stabilized the red mud by adsorption and cementation, resulting in an increase in strength and a decrease in pollutant concentration.

**Figure 15:**
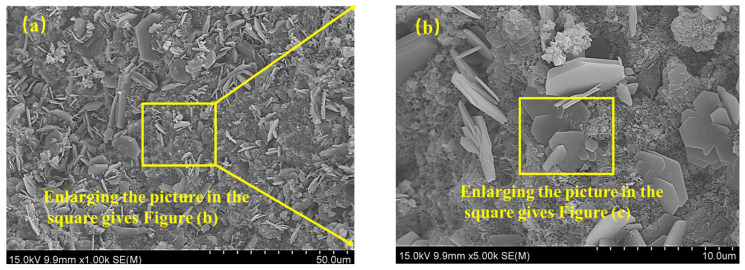

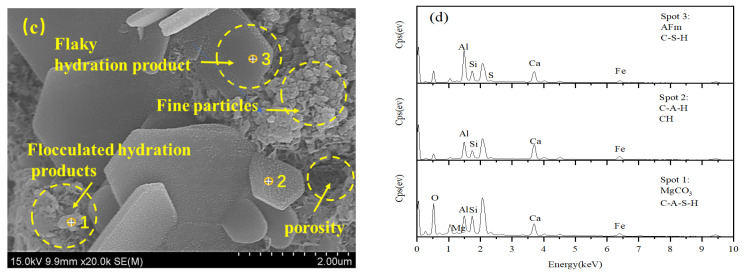
SEM-EDS of solidified/stabilized red mud after 10 freeze–thaw cycles. (**a**) ×1000; (**b**) ×5000; (**c**) ×20,000; (**d**) EDS.

Figure 15 shows the SEM-EDS of solidified/stabilized red mud after 10 freeze–thaw cycles. Compared with the specimens under standard curing conditions, after freeze–thaw cycling, a large number of grid-like hydration products were transformed into flake and petal-like products, the number of fine particles and pores increased significantly, and the integrity of the specimens deteriorated. EDS tests were carried out on the three points in the figure, in which the peaks of Mg, Al, and Si were higher at point 1, indicating that a large amount of MgCO_3_ and C-A-S-H were contained, and point 2 was a lamellar and laminar material, and contained elements such as Ca, Si, Al, indicating that Ca(OH)_2_ and C-A-H were contained in it. Point 3 was also a lamellar and petal-like material, with higher peaks of the element Al and containing S elements, indicating AFm, C-S-H, and other substances, which is consistent with the XRD results. Compared to the specimens prepared under standard curing conditions, the products of the specimens after freeze–thaw cycling showed an increase in the production of MgCO_3_ and Ca(OH)_2_ and a decrease in the production of Mg-Al-HT.

#### 3.5.3. FT-IR Results and Analysis

**Figure 16:**
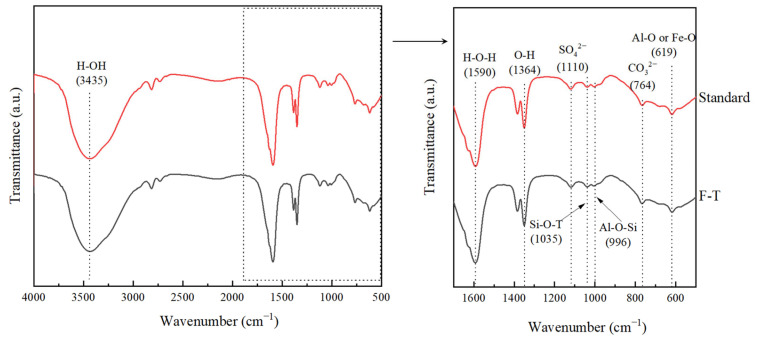
FT-IR of solidified/stabilized red mud after 10 freeze–thaw cycles.

Figure 16 shows the FT-IR of solidified/stabilized red mud after freeze–thaw cycling. The peak at 3435 cm^−1^ is the vibrational band of H_2_O, and that at 764 cm^−1^ is the vibrational band of CO_3_^2−^, indicating that CaCO_3_ crystals were generated in the products from the reaction of Ca(OH)_2_ with CO_2_ [69], and MgCO_3_ crystals were generated in the products from the reaction of Mg^2+^ with CO_2_, which is consistent with the XRD results. CO_2_ reaction produced MgCO_3_ crystals in the product, which is consistent with the XRD results. The decrease in the peak of the stretching vibration near 1590 cm^−1^ indicates a decrease in the water of crystallization produced by the reaction, which corresponds to a decrease in the alkali-excited hydration product. The peak at 1035 cm^−1^ is the vibrational band of Si-O-T (T = Si, Fe) [70,71], and that at 996 cm^−1^ is the vibrational band of Si-O-Al [72], which proves the presence of C-A-S-H gel in the products. The solidified/stabilized red mud after freeze–thaw cycling has reduced peaks and smoother curves compared to the solidified/stabilized red mud with standard curing, suggesting that the amount of C-A-S-H gel products is reduced. This is consistent with the results obtained by XRD and SEM-EDS.

#### 3.5.4. Structural Modeling of Solidified/Stabilized Red Mud

The mechanism by which CGFPA can effectively reduce the leaching concentration of pollutants in red mud is mainly divided into solidifying and stabilizing effects.

Solidification: when the CCR as well as the red mud particles in CGFPA are exposed to water, OH^−^ is decomposed to provide an alkaline environment for the hydration reaction. Under the alkaline environment, the Si-O-Si bonds, Al-O-Al bonds, and Si-O-Al bonds in the silica–oxygen tetrahedra and aluminum–oxygen tetrahedra in the volcanic ashy material GGBS as well as FA are broken to obtain reactive Si-O- and Al-O- structures, and reactive silica-aluminum-phase material undergoes a pozzolanic reaction to produce C-S-H, C-A-H, C-A-S-H, and other alkali excitation products, which improve the density of the interior of the solidified/stabilized red mud by cementing the red mud particles, filling the internal pores, etc., and thus improve the strength of the solidified/stabilized red mud. As an auxiliary material, PG decomposes SO_4_^2−^ ions in contact with water, and the following reactions occur to generate AFt (calomel) and Afm. The micro-expansion of AFt and AFm can fill the internal pores of the solidified/stabilized red mud, which further improves the degree of densification, and with the progress of the reaction, the excess OH^−^ fails to take part in the hydration reaction, and when CO_2_ in the air enters into the interior of the material through the pores, it is combined with OH^−^ to form a solidified/stabilized red mud, which is a good solution for the solidified/stabilized red mud. When CO_2_ from the air enters the interior of the material, it reacts with OH^−^ to produce the insoluble substance CaCO_3_ to fill the pores, which makes the solidified/stabilized red mud even denser.

Stabilization: OH^−^ supplied by CCR reacts with heavy metals in red mud to produce hydroxide precipitates of heavy metals. Under the action of OH^−^, depolymerization of the active silica-alumina vitrinite in FA and GGBS occurs, and the active silica–alumina material obtained by depolymerization reacts with heavy metal ions to produce silicate precipitation, and some of the heavy metals can react with SO_4_^2−^ obtained by dissolution of auxiliary material PG to produce complex salt precipitation, which stabilizes the heavy metals. Secondly, heavy metals and other cations (Na^+^, K^+^, etc.) in the alkali excitation product undergo ion exchange and are fixed inside the gel product through charge balance. The hydration product can also adsorb heavy metal ions through charge balance or intermolecular forces, and the hydration product generated by the hydration reaction simultaneously wraps the heavy metal ions inside to stabilize the heavy metals. In addition, the strong adsorption effect of graphene on heavy metal ions also effectively reduces the leaching concentration of pollutants.

**Figure 17:**
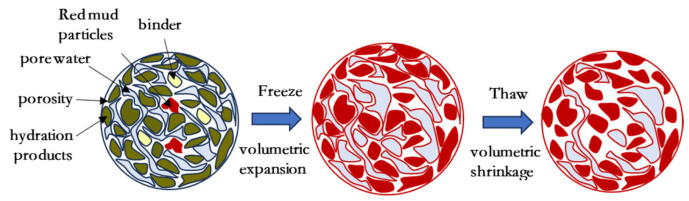
Schematic diagram of solidified red mud degradation model in freeze–thaw cycle environments.

The structural model of solidified/stabilized red mud under the action of freeze–thaw cycles can be generalized as shown in Figure 17. The internal structure of red mud becomes denser after the addition of binders. However, as the freeze–thaw cycle proceeds, ① the freeze–thaw cycle effect inhibits the alkali excitation reaction, which makes the encapsulation weaken, ② there is a change in temperature, which causes the desorption of heavy metals adsorbed on the surface of alkali excitation products and red mud particles, and ③ the agglomerates of red mud particles in solidified/stabilized red mud are deformed by thermal expansion and contraction under the action of freeze–thaw cycles, which destroys the inter-particle agglomerative structure and leads to the exposure of pollutants enclosed and wrapped inside the agglomerates to the pore water. For the above reasons, the solidifying/stabilizing effect of CGFPA binder on red mud decreases with an increase in the number of cycles, i.e., the unconfined compressive strength and pH value decrease with an increase in the number of freeze–thaw cycles, and the leaching concentration of pollutants increases with an increase in the number of freeze–thaw cycles.

## 4. Conclusions

In this paper, CGFPA-cured solidified/stabilized red mud was prepared by using calcium carbide slag (C) as the alkali exciter, ground granulated blast furnace slag (G) and fly ash (F) as volcanic ash material, phosphogypsum (P) as auxiliary material, and graphene (A) as an external dopant, and the effects of freeze–thaw cycling on the mechanical and chemical properties and leaching toxicity of the solidified/stabilized red mud were studied systematically. In addition, the damage mechanism of solidified/stabilized red mud on the macro performance was analyzed using microscopic analyses. The following conclusions can be drawn:

(1) Under the action of freeze–thaw cycles, the unconfined compressive strength of solidified/stabilized red mud decreases with an increase in the number of cycles, and the pattern is consistent with an exponential function. When the mixing ratio was 15%, 20%, 25%, and 30%, the cumulative loss rate of the solidified/stabilized red mud’s cumulative rate of loss of unconfined compressive strength was 50.6%, 47.5%, 32.2%, and 25.3%, respectively. Based on the functional failure evaluation criteria and the derived equations, it can be deduced that the number of freeze–thaw cycles for the failure of solidified/stabilized red mud is 2, 3, 16, and 26, respectively.
(2) The pH value of solidified/stabilized red mud under different mixing ratios gradually decreased with an increase in the number of cycles, the decreasing tendency gradually became slower, and the pattern satisfied an exponential function relationship.
(3) With an increase in the number of cycles, the leaching concentration of all seven pollutants increased continuously, and the logarithm of the leaching concentration and the number of cycles satisfied a linear relationship. If the leaching toxicity of the pollutants in red mud is considered, the number of freeze–thaw cycles for the failure of solidified/stabilized red mud at the mixing ratios of 15%, 20%, 25%, and 30% are 0, 2, 6, and 8 times, respectively, when the groundwater quality of Class III is used as the failure criterion.
(4) The hydration reaction of the CGFPA binder generates products such as C-S-H, C-A-S-H, C-A-H, AFm, etc. These products make the structure of solidified/stabilized red mud stronger by cementing the red mud particles and filling the internal pores and forming a grid-like interconnected structure. Under the action of freeze–thaw cycling, the lattice structure of solidified/stabilized red mud is damaged, which leads to a decrease in its strength and an increase in pollutant leaching concentration.

In this paper, red mud was taken as waste material and general industrial solid waste with calcium carbide residue, ground granulated blast furnace slag, fly ash, and phosphogypsum as components, and graphene as an external dopant was used to prepare CGFPA binder for solidifying/stabilizing the red mud. This approach can not only realize the recycling of industrial waste and achieve the purpose of “waste to waste” but also provide a theoretical basis for the large-scale utilization of red mud in the tundra region.

## Figures and Tables

**Table 1:** Physical properties of red mud.

Natural Moisture Content (%)	Specific Gravity	Liquid Limit (%)	Plastic Limit (%)	Plasticity Index
31.9	2.72	37.8	25.2	12.6

**Table 2:** Chemical composition of red mud (%).

Chemical Composition	SiO_2_	Al_2_O_3_	Fe_2_O_3_	CaO	SO_3_	Na_2_O	TiO_2_	ZrO_2_	Other
Percentage	12.66	15.79	36.41	14.98	0.86	9.61	7.34	0.61	1.74

**Table 3:** Concentration of pollutants in red mud and concentration of water quality.

Pollutants	Cu	Zn	Cr	Ni	As	Pb	Cd
Red mud	63.7	441.2	30.6	140	314	418	20.6
Water quality standard of groundwater class III	1000	1000	50	20	10	10	5

standard of groundwater class III (μg/L).

**Table 4:** Test program for solidified/stabilized red mud under freeze–thaw cycle environments.

Test Soil	Type of Binder	Mixing Ratio of Binder (%) ^a^	Total Water Content Ratio	Curing Age (d)	Number of Cycles	Test Content	Curing Environment
Red mud	CGFPA	15	1	28	0, 2, 4, 6, 8, 10	Unconfined compressive strength	Freeze–thaw cycle
						Acidity and	
						alkalinity	
		20	1.2			Toxicity leaching	
		25	1.3			XRD, SEM-EDS	
		30	1.4			FT-IR ^b^	

Note: ^a^: Binder incorporation ratio is defined as the ratio of the dry mass of binder to dry the mass of red mud. ^b^: XRD, SEM-EDS, and FT-IR measurements are conducted only for samples after 0 and 10 cycles.

**Table 5:** Table of fitted parameters.

Mixing Ratio (%)	*a*	R^2^
15	816.4	0.939
20	948.6	0.903
25	512.9	0.934
30	457.7	0.900

**Table 6:** Table of pH fitting parameter.

Mixing Ratio (%)	*b*	R^2^
15	9.93	0.995
20	10.03	0.985
25	10.20	0.999
30	10.39	0.994

## Data Availability

The original contributions presented in this study are included in the article. Further inquiries can be directed to the corresponding author.
